# Microbial Diversity in Commercial Bee Pollen from Europe, Chile, and Mexico, Based on 16S rRNA Gene Amplicon Metagenome Sequencing

**DOI:** 10.1128/genomeA.00247-18

**Published:** 2018-05-17

**Authors:** Vicente D. Moreno Andrade, Carlos Saldaña Gutiérrez, Rosa P. Calvillo Medina, Andrés Cruz Hérnandez, Moisés A. Vázquez Cruz, Alfonso Torres Ruíz, Sergio Romero Gómez, Miguel A. Ramos López, Erika Álvarez-Hidalgo, Silvia B. López-Gaytan, Natanahel Salvador Ramírez, George H. Jones, Jose Luis Hernandez-Flores, Juan Campos-Guillén

**Affiliations:** a Facultad de Química, Universidad Autónoma de Querétaro, Santiago de Querétaro, Querétaro, Mexico; b Facultad de Ciencias Naturales, Universidad Autónoma de Querétaro, Santiago de Querétaro, Querétaro, Mexico; c Escuela de Agronomía, Universidad De La Salle Bajío, León, Guanajuato, Mexico; d Departamento de Investigación y Desarrollo, KOPPERT MEXICO, El Marqués, Querétaro, Mexico; e Department of Biology, Emory University, Atlanta, Georgia, USA; f Departamento de Ingeniería Genética, Centro de Investigación y de Estudios Avanzados del IPN, Irapuato, Guanajuato, Mexico

## Abstract

Bee pollen is a highly nutritive natural foodstuff. Because of its use as a comestible, the association of bacteria with bee pollen is commercially and biologically important. We report here the bacterial diversity of seven bee pollen samples (five from Europe, one from Chile, and one from Mexico) based on 16S rRNA gene amplicon metagenome sequencing.

## GENOME ANNOUNCEMENT

Bee pollen has for years been touted as a highly nutritious food (1). It is also used in the rearing of insects of agricultural interest, such as honey bees (Apis spp.), bumblebees (Bombus spp.), and syrphids (Sphaerophoria spp.) (2, 3). Therefore, the commercial demand for bee pollen by beekeepers has increased in recent years. Countries, such as Spain, China, Australia, and Argentina, are the main producers of bee pollen (4). One issue which affects the utility of bee pollen as a human foodstuff and in the commercial context is the association of microbes with the pollen. The structural and nutritional characteristics of bee pollen, as well as the potential for contact with the environment and with other insects during production, make bee pollen a favorable microhabitat for many microorganisms (5). Microbial diversity in pollen samples has been studied in specific areas (6). The objective of the present study was to analyze the bacterial diversity present in commercial bee pollen from Europe, Chile, and Mexico.

Bee pollen samples were obtained from three different suppliers. We worked with seven such samples, five samples from Europe, one sample from Chile, and one sample from Mexico. DNA extraction for each sample was carried out with a ZymoBIOMICS DNA minikit (Zymo Research, Irvine, CA, USA). Amplicons of the region V3-V4 for 16S rRNA genes were amplified with primers 337F/805R (7) using the Illumina MiSeq platform at Macrogen, Inc. (Seoul, Republic of Korea). The total numbers of raw sequences were 179,758 to 214,249 for the 16S rRNA gene libraries. The sequences were subjected to low-quality trimming, merging of paired reads, and elimination of reads shorter than 50 bp. To identify operational taxonomic unit (OTUs), from 122,465 to 151,548 filtered reads from 16S rRNA gene metagenomic libraries were clustered, and the processed sequences were uploaded to the service cloud for the analysis of bacterial diversity with the 16S Biodiversity Tool and classified with RDP Tools version 2.12 (8, 9), with 97% identity. Bioinformatic analysis was carried out using Geneious software version 9.

Based on the amplicon analysis, the most prevalent phylum in all samples was the *Cyanobacteria*, with a relative abundance of 50% to 76%. The relative abundances of other phyla were 12% to 28% for *Proteobacteria*, 1% to 3% for *Thermotogae*, and 1% to 37% for *Firmicutes*. Bacterial orders with a relative abundance of greater than 1% were as follows: in the sample from Chile, *Rhodobacterales* (10%), *Rhodospirillales* (8%), *Rickettsiales* (1%), *Lactobacillales* (2%), and *Petrotogales* (1%); in the sample from Mexico, *Rhodospirillales* (5%), *Rhodobacterales* (4%), *Rickettsiales* (2%), *Pseudomonadales* (1%), *Lactobacillales* (10%), and *Petrotogales* (2%); and in the samples from Europe, *Lactobacillales* (4% to 37%), *Rhodobacterales* (6% to 18%), *Rhodospirillales* (3% to 8%), *Pseudomonadales* (1% to 3%), *Petrotogales* (1% to 3%), and *Rickettsiales* (1% to 2%).

These results extend our knowledge of the diversity of bacteria associated with bee pollen and may be useful in developing procedures for microbial control during pollen production.

### Accession number(s).

The sequences obtained in the present study were deposited in the Sequence Read Archive (SRA) via the National Center for Biotechnology Information (NCBI) under the numbers SRP132301 (Chilean sample), SRP132302 (Mexican sample), and SRP132303 (European samples, SRX3654272, SRX3655212, SRX3655293, SRX3655335, and SRX3655336).

## References

[1] Feás X, Vázquez-Tato MP, Estevinho L, Seijas JA, Iglesias A 2012 Organic bee pollen: botanical origin, nutritional value, bioactive compounds, antioxidant activity and microbiological quality. Molecules 17:8359–8377. doi:10.3390/molecules17078359. 22785265 PMC6268883

[2] Amorós-Jiménez R, Pineda A, Fereres A, Marcos-García MÁ 2012 Prey availability and abiotic requirements of immature stages of the aphid predator *Sphaerophoria rueppellii*. Biological Control 63:17–24. doi:10.1016/j.biocontrol.2012.06.001.

[3] Velthuis HW, Van Doorn A 2006 A century of advances in bumblebee domestication and the economic and environmental aspects of its commercialization for pollination. Apidologie 37:421–451. doi:10.1051/apido:2006019.

[4] Estevinho LM, Rodrigues S, Pereira AP, Feás X 2012 Portuguese bee pollen: palynological study, nutritional and microbiological evaluation. Int J Food Sci Technol 47:429–435. doi:10.1111/j.1365-2621.2011.02859.x.

[5] Crovadore J, Gérard F, Chablais R, Cochard B, Jensen KKB, Lefort F 2017 Deeper insight in beehives: metagenomes of royal jelly, pollen, and honey from lavender, chestnut, and fir honeydew and epiphytic and endophytic microbiota of lavender and rose flowers. Genome Announc 5:e00425-17. doi:10.1128/genomeA.00425-17. 28572315 PMC5454198

[6] Lozo J, Berić T, Terzić-Vidojević A, Stanković S, Fira D, Stanisavljević L 2015 Microbiota associated with pollen, bee bread, larvae and adults of solitary bee *Osmia cornuta* (Hymenoptera: Megachilidae). Bull Entomol Res 105:470–476. doi:10.1017/S0007485315000292. 25895542

[7] Klindworth A, Pruesse E, Schweer T, Peplies J, Quast C, Horn M, Glockner FO 2013 Evaluation of general 16S ribosomal RNA gene PCR primers for classical and next-generation sequencing-based diversity studies. Nucleic Acids Res 41:e1. doi:10.1093/nar/gks808. 22933715 PMC3592464

[8] Wang Q, Garrity GM, Tiedje JM, Cole JR 2007 Naive Bayesian classifier for rapid assignment of rRNA sequences into the new bacterial taxonomy. Appl Environ Microbiol 73:5261–5267. doi:10.1128/AEM.00062-07. 17586664 PMC1950982

[9] Kearse M, Moir R, Wilson A, Stones-Havas S, Cheung M, Sturrock S, Buxton S, Cooper A, Markowitz S, Duran C, Thierer T, Ashton B, Meintjes P, Drummond A 2012 Geneious basic: an integrated and extendable desktop software platform for the organization and analysis of sequence data. Bioinformatics 28:1647–1649. doi:10.1093/bioinformatics/bts199. 22543367 PMC3371832

